# Do global health networks facilitate innovation, learning and sharing? A qualitative analysis of the Quality-of-Care Network in Bangladesh, Ethiopia, Malawi, and Uganda

**DOI:** 10.1371/journal.pgph.0002720

**Published:** 2025-01-29

**Authors:** Kondwani Mwandira, Seblewengel Lemma, Albert Dube, Kohenour Akter, Asebe Amenu Tufa, Agnes Kyamulabi, Gloria Seruwagi, Catherine Nakidde, Kasonde Mwaba, Nehla Djellouli, Charles Makwenda, Tim Colbourn, Yusra Ribhi Shawar

**Affiliations:** 1 Parent and Child Health Initiative Trust, Lilongwe, Malawi; 2 Department of Disease Control, London School of Hygiene & Tropical Medicine, based at the Ethiopian Public Health Institute, Addis Ababa, Ethiopia; 3 Perinatal Care Project, Diabetic Association of Bangladesh, Dhaka, Bangladesh; 4 Ethiopian Public Health Institute, Addis Ababa, Ethiopia; 5 Department of Social Work and Social Administration, Makerere University School of Public Health, Kampala, Uganda; 6 Institute for Global Health, University College London, London, United Kingdom; 7 Bloomberg School of Public Health, Johns Hopkins University, Baltimore, United States of America; 8 Paul H. Nitze School of Advanced International Studies, Johns Hopkins University Washington, DC, United States of America; University of California San Francisco, UNITED STATES OF AMERICA

## Abstract

The Quality-of-Care Network (QCN), launched by WHO and partners, links global and national actors across several countries to improve maternal and newborn health. We conducted a prospective qualitative study to examine how QCN in Bangladesh, Ethiopia, Malawi and Uganda facilitated learning, sharing, and innovation within and between network countries. We conducted 227 key informant interviews with QCN actors at global, national, and facility levels iteratively in two to four rounds from June 2019 to March 2022. We also reviewed all accessible QCN documents. Drawing on knowledge sharing theory, we thematically analysed the qualitative data according to three themes: sharing, learning, and innovations. Sharing and learning were evident through virtual and in-person platforms including conferences and webinars, held on online resource libraries such as the QCN website. This provides access to strategies and approaches shared by countries and actors. Locally, there was a strengthening of learning collaborative meetings, coaching, and mentorship. Regular meetings, such as stakeholder coordination meetings and learning collaborative sessions, provided opportunity for stakeholders to strategize, share and learn maternal and child health approaches. The network also promoted coordination among stakeholders. Common sharing and learning approaches, such as learning collaborative sessions, were evident across QCN countries. However, innovation was not as apparent across countries. While there were some exceptions, such as the development and adoption of innovative software applications aimed at boosting the capacity of service providers in network countries, these were limited. Most innovation approaches were similar to pre-existing maternal health approaches, adopted from an era preceding the QCN. Nevertheless, there was evidence that QCN improved their functionality. We provide evidence of how learning, sharing, and innovation among and within countries can be fostered for improving maternal and child health; and limitations. This understanding may help country efforts to achieve targets for ending preventable maternal and neonatal deaths.

## Introduction

Many women and newborns continue to die from complications in pregnancy and childbirth due to poor quality and access to peripartum care [1]. In 2017, maternal and newborn deaths were estimated at 295,000 and 2.5 million respectively, with Sub-Saharan Africa accounting for 66% and 41% of those deaths respectively [2–5]. While rates of institutional delivery have increased and effective interventions exist to treat the main causes of maternal and neonatal deaths in low and middle-income countries (LMICs), effective implementation of quality care remains a challenge [6,7]. Inadequate quality care is mostly driven by insufficient human resources, poor training of health staff, inadequate infrastructure, shortages in equipment, and cultural beliefs at the community level [8,9]. Furthermore, postnatal check coverage is also a huge problem in LMICs; only about 60% of newborns in LMICs receive postnatal checks within 48 hours of delivery, which is key for early identification of complications and prompt care [4].

In response to the challenges faced in the implementation of quality care across many LMICs, the World Health Organization (WHO) and global partners launched ‘*The Network for Improving Quality of Care for Maternal, Newborn and Child Health*’ (QCN) in 2017 [10,11,12]. Initially, the network comprised nine countries: Bangladesh, Cote d’Ivoire, Ethiopia, Ghana, India, Malawi, Nigeria, Tanzania and Uganda, with Sierra Leone joining later in 2017, and Kenya joining in 2019. This study was conducted in Bangladesh, Ethiopia, Malawi, and Uganda. We selected these four countries because they represent a range of starting points and contexts. The network aimed to build a cross-country platform to exchange information and improve maternal and child health. Furthermore, it also aimed to facilitate joint learning across health facilities and countries around quality improvement implementation approaches based on a shared theory of change (ToC) and health outcome goals. Through these efforts, the goal was to reduce in-facility maternal and child death case fatality rates by 50% in a five-year period (2017–2022).

**Fig 1:**
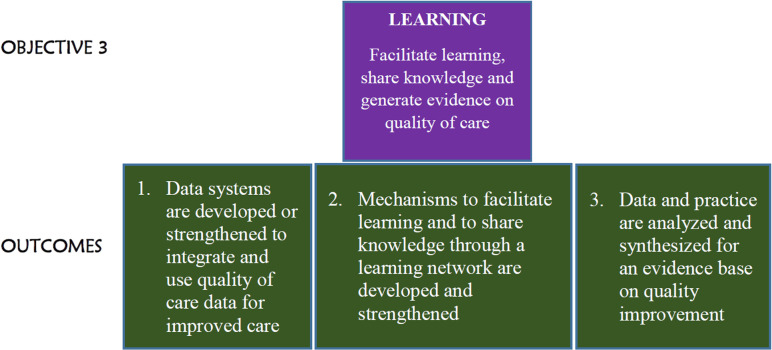
LALA framework strategic objective 3.

The QCN was guided by four strategic objectives cited in the WHO’s quality of care framework called “Learning, Accountability, Leadership and Action” (LALA) [13]. The third objective (learning) focused on three prominent outcomes: (1) data systems development or strengthening to integrate and use quality of care data for improved care; (2) development and strengthening of mechanisms to facilitate learning and sharing of knowledge through a learning network; (3) analysis and synthesis of data and practice for an evidence base on quality improvement (Fig 1). These objectives were developed on the assumption that individual countries and the global community have much experience of what works and does not work, and each country needs to adapt solutions to its local context. Therefore, it was presumed that a learning network would foster the free transfer of information and knowledge between and within all countries interested in improving quality of care.

Accordingly, this paper examines QCN facilitation of learning, sharing and innovation, within and between network countries. Specifically, we analyze how the attributes of the network, as well as its operational strategy and performance affect learning, sharing, and innovations among network actors at the global and national level. Given LMIC impetus to achieve Sustainable Development Goal (SDG) targets for ending preventable maternal and neonatal deaths [14], understanding the quality and extent of learning, sharing, and innovation that takes places across LMICs on quality of care is critical.

## Methods

We conducted a prospective, iterative qualitative study (i.e., our study was planned in advance and involved multiple rounds of qualitative data collection, each building on the previous round) to analyse the QCN in Bangladesh, Ethiopia, Malawi, and Uganda. This analysis was part of a broader multi-country evaluation of QCN [15–21] (S1 Text). Common methods are reported in our methods supplement for our QCN evaluation collection of papers (S2 Text). Data collection in Malawi began on September 14, 2019 and ended on March 31, 2022; in Bangladesh, began on June 4, 2019 to March 31, 2022; in Ethiopia, began on May 15, 2020 to March 31, 2022; and in Uganda, began on February 19, 2021 to March 18, 2022. The key aspects of the methods used in this study are summarized below.

### Research setting and population

This research was conducted in Bangladesh, Ethiopia, Malawi, and Uganda; these were the selected case study countries in our broader QCN Evaluation Study given their variance in starting points on addressing maternal and child mortality, as well as their political context differences (S1 Text). We conducted key informant interviews (KIIs) with purposively selected individuals representing each stakeholder group, including the Ministry of Health, implementing partners, and local and international non-governmental organisations at the national, district, and facility level. KIIs were conducted at the facility level in four QCN-selected facilities in each country. These four facilities were selected at the onset of the study in 2019 and were a subset of a wider group of facilities selected by each country’s respective Ministry of Health to spearhead the implementation of quality of care network interventions [15]. These facilities were referred to as QCN learning facilities. The four selected facilities included two high and two low performing (Table 1). This performance-based selection was based on maternal and new-born health outcomes at the onset of the QCN (data in 2017) and after a few years of QCN implementation (data from 2019 at the onset of the evaluation). We then compared maternal and child mortality indicators and isolated two high-performing and two low-performing facilities from a wider group of learning facilities for each country. We did this to maximize variation in our observations, given the purposive nature of our qualitative investigation.

**Table 1:** Selected study facilities.

Country	High performing	Low performing
**Bangladesh**	Case 1 = District Hospital 1	Case 2 = District Hospital 2
	Case 3 = Upazila Health Complex 1	Case 4 = Upazila Health Complex 2
**Ethiopia**	Case 2 = Health Centre2	Case 3 = General Hospital 1
	Case 4 = Referral Hospital 1	Case 1 = Health Centre 1
**Malawi**	Case 2 = District Hospital 2	Case 1 = District Hospital 1
	Case 4 = Central Hospital 1	Case 3 = District Hospital 3
**Uganda**	Case 1 = Hospital 1 = high performing	Case 3 = Hospital 3
	Case 2 = Hospital 2 = high performing	Case 4 = Health Centre IV

### Data used

#### Key informant interviews.

We conducted semi-structured interviews with selected national and local level network members and key stakeholders in Bangladesh, Ethiopia, Malawi, Uganda, and the QCN Secretariat between October 2019 and May 2022. At the global and national level, we interviewed actors from the Ministry of Health (MoH) and key implementation partners, such as the Bill and Melinda Gates Foundation, WHO, UNFPA, UNICEF, and GIZ. Beyond the national level, we conducted qualitative interviews with key quality improvement and QCN representatives in 4 selected district facilities in each country (Table 1). These representatives included district health officers, district quality improvement officers, safe motherhood coordinators, medical superintendents, maternity in-charges, and frontline healthcare workers. Particular attention was paid to the perspectives and goals of those carrying out the work of the network, with a specific interest in information pertaining to learning and sharing platforms within the network and pertinent innovations linked to the delivery of care in facilities, and innovations in delivery of strategic management. Two to four rounds of key informant interviews were conducted in study countries to capture the evolution of network operations on innovations, learning and sharing, as well as views pertaining to network activities. The iterative nature of our study included follow-up on emerging findings from the previous rounds. In total, 227 KII were conducted at the country level (Table 2).

**Table 2:** Number of qualitative interviews conducted.

Country	Number of interviews	Dates conducted	
		Start	End
**Bangladesh**	75	October 2019	March 2022
**Ethiopia**	40	January 2021	November 2021
**Uganda**	55	November 2020	March 2022
**Malawi**	57	October 2019	March 2022
**Total**	**227**		

#### Document review.

We reviewed all accessible published documents, unpublished documents and communications relating to QCN. These included strategy and management documents, operational plans, directives, formal minutes, and reports (Table 3). To ensure the credibility and reliability of these documents, we reviewed documents from official websites only, especially those from QCN website [13,22], and government, WHO, and UNICEF websites [1,10,23]. We accessed unpublished documents (strategy and management documents, operational plans, directives, formal minutes, and reports) through our WHO and MoH QCN contacts. We also reviewed accessible virtual materials (such as webinars and seminars) from websites and YouTube channels specific to the QCN.

**Table 3:** QCN document reviews completed.

QCN evaluation level	Document type	Number of documents reviewed
**Global**	Strategy document	5
	Operational plan	5
	Report	5
	Minutes	0
**National - Bangladesh**	Strategy document	3
	Operational plan	1
	Report	5
	Minutes	35 (8 presentations)
**National – Ethiopia**	Strategy document	4
	Operational plan	6
	Report	7
	Minutes	20
**National – Malawi**	Strategy document	1
	Operational plan	2 (1 presentation)
	Report	9 (2 presentations)
	Minutes	6
**National – Uganda**	Strategy document	3
	Operational plan	2
	Report	9
	Minutes	0

### Data analysis

To examine QCN facilitation of learning, sharing, and innovation, we analysed the qualitative data (KIIs and documents) for each of the four countries and global-level data across all rounds as separate entities. We synthesized and populated country-specific qualitative data separately from all data collection rounds, and then analysed according to three common themes (Table 4).

**Table 4:** Data population framework – themes.

1. Sharing	2. Innovations	3. Learning
Knowledge Management Knowledge transfer Diffusion of innovation Social systems-adopter’s category	Tools of knowledge management	Knowledge Management Diffusion of Innovations Network Organization and Effectiveness Knowledge Utilization Factor influencing adoption

**Fig 2:**
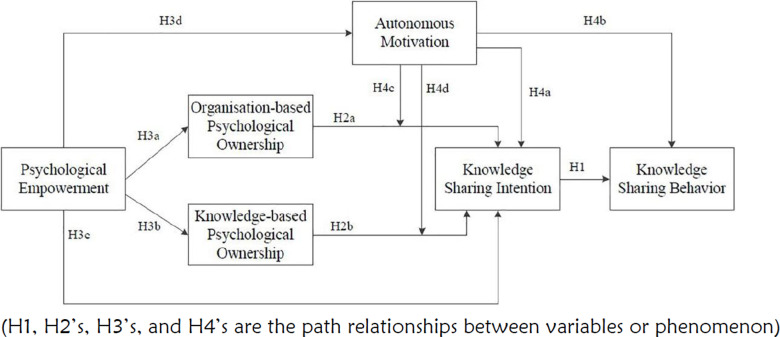
Learning and sharing theory.

To understand factors that drive and influence knowledge sharing and learning in the global context across countries, we used a common coding framework (codebook) developed based on a theory adopted from Wu SY et al 2014 [24], “*Knowledge Sharing Among Healthcare Practitioners: Identifying the Psychological and Motivational Facilitating Factors*” (Fig 2) to organise and present the results.

The theory hypothesizes that knowledge sharing behaviour (KSB) is a product of knowledge sharing intention. The theory suggests that knowledge sharing intention is directly influenced by psychological empowerment (path H3e; Fig 2) or indirectly through intermediate predictors such as organization-based psychological ownership (path H3a; Fig 2), knowledge-based psychological ownership (path H3b; Fig 2), and autonomous motivation (path H3d; Fig 2). We drew on the theory to deductively code the data, and to organize and present global results on psychological empowerment and psychological ownership (organization-based and knowledge-based) as direct and intermediate predictors respectively, that influence knowledge sharing intention. While the data was deductively coded drawing on this theory, we also inductively identified new codes that were deemed relevant (S2 Text), in order to represent and interpret the data as accurately as possible.

Psychological empowerment focuses on personal perceptions and empowering experiences, and how organizations build a conducive environment for these. Organization-based psychological ownership can be built via knowledge sharing behaviours, improved teamwork, communication and coordination, and can facilitate changes in behaviour that can improve practices in quality care service delivery.

We also conducted a thematic desk review of accessible documents (Table 3) and online resources such as websites and YouTube channels associated with QCN. We focused on themes around learning, sharing and innovations, how they have been articulated, what they intend to achieve, and how they have been planned to be scaled out to countries and facilities. All quotes are identified by the data collection round (e.g., Round 1), the level of data collection (e.g., national and local), the country, and the type of stakeholder interviewed (e.g., Ministry of Health; implementation partner; hospital health worker). At the national level, the type of stakeholder is not given as there are few of each type and we need to preserve anonymity. We used NVivo 12 software to organize our qualitative analysis.

### Ethics

All interviews were conducted after obtaining informed written consent from participants, including separate consent for tape recording. All data is confidential and anonymized. Ethical approval was obtained from the Research Ethics Committee at University College London (3433/003), the National Health Sciences Research Committee in Malawi (Protocol number: 19/03/2264), the Institutional Review Boards in Bangladesh (BADAS-ERC/EC/19/00274), the Ethiopian Public Health Institute in Ethiopia (EPHI-IRB-240-2020) and Uganda (Makerere University School of Public Health Higher Degrees Research Ethics Committee, Ref: 869). Additional information regarding the ethical, cultural, and scientific considerations specific to inclusivity in global research is included in the Supporting Information (S1 Checklist).

## Results

We present global level results systematically under sharing and learning themes adopted from a learning and sharing theory by Wu et al 2014. Foremost, we present results on psychological empowerment, being the first main driver of learning and sharing intents, and psychological ownership (organization and knowledge-based) being the intermediate predictor of learning and sharing intentions and behaviour. We further explain how these influence learning and sharing across member countries at the global level. We also present a narrative of findings on platforms available globally for learning and knowledge sharing. We then present narrative results on learning, innovation and sharing for each of the four countries included in our study, Malawi, Bangladesh, Uganda, Ethiopia, in turn. This representation is also in line with LALA strategic objective number 3: mechanisms to facilitate learning and to share knowledge through a learning network (Fig 1). We end with an analysis of key common implementation challenges across the countries.

### Global level

#### Psychological empowerment.

QCN’s establishment was largely motivated by the observation that different countries were approaching issues around maternal and child health in different ways, and that countries could benefit from an information sharing approach. QCN actors from global and national levels indicated QCN to be key in the facilitation of knowledge sharing on quality-of-care practices among countries. QCN allowed countries to learn and adopt working strategies and practices from other countries with similar contextual factors. Sharing of strategies was also perceived to be a medium for finding common solutions to address causes of maternal and child mortality that are prevalent in LMICs, such as sepsis, birth asphyxia, and other childbirth complications [25]. One respondent shared the value in exchanging learnings across countries:

*“Sharing of information...is important for learning. The problem we are dealing with in our country might have been dealt with by some other country. Maybe that other country addressed that problem successfully, which can be used to solve our country’s problem. Then, some learning about our country may be helpful to other countries.”* ***Round1-National-Bangladesh***

One major component of psychological capability for QI in QCN member countries like Bangladesh was adequate training and reporting systems, which enabled improved monitoring, as well as learning from identified deficiencies. QCN-related activities in Bangladesh, particularly the efforts of UNICEF, Save the Children-IHI, and QIS, focused on introducing new data systems and training hospital staff to use them. Global level webinar training for national level actors also acted as a tool for psychological empowerment for in-country actors to spearhead implementation.

#### Psychological ownership; knowledge-based and organisation-based.

The operational design of the QCN fostered network ownership and motivated in-country actors to share and learn. Specifically, QCN’s enactment enabled actors at the national and facility level to take the QCN activities implementation lead and champion implementation independently. At the same time, QCN’s design ensured that global and national actors oversaw implementation at the national, district and facility levels. For instance, in Malawi and Bangladesh, local quality improvement focal persons (district and facility QI coordinators) and Quality Improvement Support Teams (QISTs)-sitting below the QCN coordination team at the national level- were selected within their implementation level to oversee QCN activities implementation within their districts and facilities, in turn, this arrangement helped foster a sense of ownership. QIST, for example, which operated at district and facility management level comprising heads of departments, was responsible for overseeing quality improvement in their respective districts and health facilities.

In terms of reporting hierarchy, QIST reported to a quality improvement focal person within their district and facility, below the QI focal persons, each MNCH ward had its own Work Improvement Team (WIT), which was responsible for implementing ward level QI projects to address ward related QI issues, largely informed by QI data and indicators over a specified period. The national level stakeholder’s major responsibility was to provide strategic support, while implementation was done by local teams to ensure local ownership. For instance, in Malawi, the quality improvement plans developed by the WITs outlined the QI efforts they undertook based on the needs and gaps identified through the baseline assessments facilitated by the Quality Management Directorate (QMD). This in turn encouraged high participation by QISTs and WITs representatives at national level collaborative meetings and other knowledge-sharing events, as described by one respondent:

*“The fact that the institutions can identify problems is one result in itself. The development of quality projects, identifying problems, solving those problems and sharing lessons with others is a manifestation in itself. Designing the projects, implementing and reporting for the federal government and the federal government to make them available for others to share experiences is a great manifestation”* ***Round1-facility-Ethiopia***

### Sharing and learning platforms

#### Virtual and in-person meetings for sharing and learning within the QCN.

Globally, the network offered several platforms for rapid sharing across its member countries, with the ability to bring high-level professionals from different countries and regions together to discuss key issues relevant to the network. This was centrally organized by the WHO and UNICEF. Countries were brought together through virtual forums like webinars and conference calls. Where feasible, face-to-face meetings were also conducted. Webinars had been conducted since the onset of the QCN in 2017, and they became more prominent in 2020 as the onset of the COVID-19 pandemic stalled regular face-to-face meetings between network members. Webinars ultimately proved to be much less costly compared to face-to-face meetings, which required significant resources to bring people together.

One prominent example of a virtual meeting was the WHO-coordinated meeting called “*Integrated approach for implementing MNCH-QOC* [Maternal, Newborn, and Child Health-Quality of Care]*”,* conducted in August 2020, where Malawi and Uganda, along with other member countries, were in attendance. During this meeting, experts from the WHO and partners led the mentoring of countries on several issues, such as strategies to ensure sustained quality care for clients in health facilities. Prominent face-to-face meetings include the March 2019 meeting in Addis Ababa, which had French-English bilingual translation services to allow the participation of Francophone countries. The meeting provided both plenary and focused small group opportunities for the 11 network countries and 11 observer countries to share their experiences and progress in working towards improved quality of care.

#### Website-based platforms for sharing, learning and accessing QCN resources.

Dedicated online websites were also created to serve as a key resource for all network related material [13]. For instance, a QCN-dedicated YouTube channel was established in March 2019, where several webinars were posted. As of September 15, 2023, the channel had 695 subscribers, 102 videos posted, and 29079 views [26]. The webinars posted mainly served as a refresher for the “*WHO MNCH Quality of Care Guidelines*” and a space for countries to share implementation progress.

From the webinar series available on the QCN dedicated website, a notable few data-oriented innovations were evident. For example, an online application called “Safe Delivery App” [27], was shared by stakeholders from Ethiopia in 2020. Developed together with healthcare workers, the application was designed to provide service providers with evidence-based and up-to-date clinical guidelines on the most common childbirth procedures through animated instructions and videos. It covers the most common pregnancy and childbirth-related complications based on international maternal health guidelines [27–29]. Countries including Bangladesh, Guinea, Ghana, and India adopted the customized version of this application. In India, for example, from 2018 to 2021, there have been more than 100,000 downloads of the application; 86,242 active application users employ it as a job aid or self-learning tool. It is used in 698 districts across all 36 states and Union Territories (UTs) [30].

The QCN dedicated website was designed with multiple functionalities that, among others, would enable country actors to explore practical resources such as all QCN updates and reports. By grouping updates, reports and other webinars by country, the “*learn from other countries section*” also provided an opportunity for country actors to explore country specific QCN material.

### Country-specific learning, innovation, and sharing

#### Malawi.

At the national level, a QCN coordination team was established in 2019. Its operations were coordinated by the Ministry of Health’s Quality Management Directorate (QMD) with a dedicated officer responsible for stakeholder coordination [20]. With funding from partners such as WHO, UNICEF, GIZ, and USAID, regular meetings were held by this committee, among others, to review and develop strategic documents, brainstorm quality improvement implementation progress and provide strategic planning and technical support to the network. Between 2017 and 2021, the stakeholder’s coordination team met quarterly, and sometimes at monthly non-regular intervals depending on the availability of resources to conduct these meetings and urgent needs. A respondent described the coordination mechanisms implemented in Malawi:

*“In terms of coordination at the national level, there is a coordination team that comprises partners as well as the government, which meets almost on monthly basis just to discuss how the whole network in terms of performance and implementation how it is progressing... it meets on monthly basis”* ***Round1-National-Ministry of Health-Malawi***

Key outcomes of these coordination meetings included strategic documents such as the “*Maternal New-born Child and Adolescent Health Quality of Care Roadmap*” [31] and “*The Malawi MNH Standards*”[32] . These and other QCN strategies were shared with learning facilities through Trainings of Trainers (ToTs) led by national level stakeholders. These ToTs built local capacity and created ownership to ensure sustainability. Those trained took the lead in teaching their fellows in their facilities.

Beyond the national level, learning and sharing were carried out through quarterly collaborative learning sessions at the zonal and district levels. Learning facilities came together to share and showcase various QI project approaches and successes being carried out in their respective MNCH wards. Facilities also presented their quantitative data on how they have performed on reducing in-facility child and maternal mortalities. Those doing better in one aspect shared what they were doing to attain such an achievement so that others could learn and adopt. Facilities doing poorly also shared challenges during these sessions to discuss possible solutions. A respondent discussed how facilities shared their learnings with one another:

*“Ok, in these meetings we normally discuss the indicators on how we are faring on maternal and neonatal indicators. So, we meet like clusters; we group these health facilities in clusters where we meet for some hours and each facility presents their data on how they have performed in antenatal, labor delivery, postnatal. From there, we discuss and others learn from other facilities who are doing better. Others facilities which are not doing better, they tell us their problems as to why they are not doing better in that part and we help each other coming up with solutions to address the challenges.”* ***Round2-National-Ministry of Health-Malawi***

In some districts, learning sites were brought together to share notes on progress and challenges on a quarterly basis. Beyond these learning sites, implementation partners also supported integrated collaborative sessions between quality-of-care learning sites and the other sites within the district so that the learning sites could lead and showcase what they are doing for other facilities to learn, as noted by a respondent:

“*We made sure to bring together these learning sites so that we could share notes on a quarterly basis… notes on the progress and challenges and then we also used to support the integrated collaborative learning sites, where we had now the quality-of-care learning sites versus the other sites. So, we could bring all the health centers together at the cluster level, taking into consideration that those which are the learning sites could now lead and showcase what they are doing so that others can learn. That was the key initiative that we were doing*………….” ***Round3-National-Implementation Partner-Malawi***

#### Bangladesh.

Since early 2020, several learning and sharing platforms within the QCN were evident in Bangladesh. These included national webinars for digital trainings for hospital staff, WhatsApp groups between various district staff, virtual group calls between district officials, and in-person meetings between facility level staff. Bangladesh’s internal quality improvement system had a long-term focus on information sharing between various districts and facilities. Respondents reported engaging regularly using both national and international channels of communication, including through webinars, poster presentations, and newsletters. These channels of communication proved more useful and prominent as the COVID-19 pandemic progressed in 2020, enabling QCN members to adapt to the restrictions posed by the pandemic. One respondent discussed how learning practices shifted during the pandemic:

*“In pre-COVID period, we had some activities on knowledge sharing, one activity was across the facility learning visit, also called experience sharing visit, so that’s what we did among our QED* [Quality, Equity, Dignity; another name for the Quality of Care Network] *facilities. Staff of new four facilities visited old facilities. So, we did not have to provide so many trainings there because we found that [facility visit] was very effective. They visited those facilities and they replicated those quickly. So that’s what we did before COVID. During COVID, through these virtual platform and webinar, we shared learning and practices with each other.”* ***Round2-National-Implementation Partner-Bangladesh***

As of June 2021, one project operating in 86 hospital facilities, including some learning sites, introduced quality improvement committees and ward improvement teams in health facilities. It also established district-level learning networks across these teams for sharing strategies and progress in QI initiatives. As part of its planning, the project explicitly intended to facilitate “cross-learning” within the QCN.

Several measures have been implemented to support knowledge management in Bangladesh. By November 2021, the National Institute of Preventive and Social Medicine (NIPSOM) was developing its skills and strengthened its systems to support QI efforts in the country. They developed “QI coaches”, who in turn provided hands-on support to facilities in using QI methods, building systems to disseminate lessons on successes and failures on efforts to improve quality of care, and building capacity to manage QoC data. The nature of knowledge transmitted through the QCN network in Bangladesh mainly focused on best practices and relatively organic conversations with national-level leaders from other countries, as one respondent described:

*“Since there was no history of quality improvement activities in one hospital, one partner had to start with the basics including introducing staff to QI approaches, human resource management, and mentorship. They supported this through various technical training as well as first-hand learning, e.g., PDCA* [Plan-Do-Check-Act] *as well as learning sessions”* ***Round2-National-Implementation Partner-Bangladesh***

#### Uganda.

With the arrival of COVID-19 in 2020, online platforms like Zoom and webinars provided an ideal space for sharing and learning within the QCN in Uganda for national and facility-level actors. Learning facilities interfaced with the national level actors through Zoom meetings to share their QCN implementation experiences. Respondents found Zoom meetings useful, as they cost effectively learnt what other facilities were doing. Online platforms, such as WhatsApp groups were also found to be feasible, innovative, and cost-saving means for closing the communication gap between national and facility-level actors. Virtual platforms provided a similar environment to face-to-face meetings and trainings at a sustainable cost.

Implementation partners at both the national and district levels put together guidelines for QoC to train health workers and support the learning and sharing processes. At the local level, health workers continued to learn and seek ideas from other QCN facilities. Departmental Continuous Medical Education (DCME) was responsible for bringing Antenatal Clinic (ANC) improvement teams together. Those within the facilities shared challenges, identified gaps, agreed on recommendations and action plans, and later assigned a responsible person to ensure the action plans were implemented. Collaboration links also existed between the ANC and the maternity teams at the same facility. Maternity heads relayed their duty roster to the ANC so that the ANC could inform them of the person on duty at the right time. One of the most notable aspects learned by frontline healthcare workers in Uganda was the usage of oxygen systems and case management – a huge gap prior to the QCN:

*“I have noticed that this initiative would work better because many of the health workers didn’t know how to operate oxygen systems, so in line with QCN, I would say yes, oxygen therapy and case management using oxygen therapy is one of the initiatives that would work better to improve QI”* ***Round1-National-Uganda***

Training manuals using the Quality-of-Care training packages for MNH and simplified QI guides for facilities implementing MNH standards were developed at the national level. In 2021, participants indicated plans to have a national-level space to gather the implementers of QCN to learn how to make improvements. They identified implementation success to be linked to the QCN. While the change in leadership and organization had facilitated more regular meetings and engagement between partners, this success was yet to be fully organized, documented, and synthesized for sharing to be put into practice, as noted by one respondent:

*“I think that one of the important things that is supposed to show that the network is maturing is if learning evidence is being effectively generated, shared and used for implementation. So, if you ask me whether that is happening, I will say yes. If you ask me whether that is happening in an organized manner, I will not be very sure about what to tell you.”* ***Round1-National-Uganda***

#### Ethiopia.

In Ethiopia, at the national level, notable sharing and learning platforms, such as Telegram channels, were used for quality improvement work in the regions where MNCH projects were being implemented. QCN-specific works were also posted on the same group for the wider MNCH group to appreciate. Regional level meetings and learning collaborative sessions also provided a platform for strengthening sharing and learning.

In regional meetings, stakeholders shared experiences from the implementation of the QCN. During these meetings, learning facilities were given the opportunity to present their QI projects, and presenting facilities were advised by experts from the national level on areas to improve and maintain. Rewards and recognition were given to well performing facilities.

Learning collaborative sessions and coaching were also available at the local level for sharing the best quality improvement projects. Usually conducted for a period of three days, the hospitals and health centers in the region presented the best practices in QI projects, providing an opportunity for learning and addressing challenges. Coaching programs, which occurred once or twice a year, also provided a learning opportunity for the frontline healthcare workers. Although supportive supervision was conducted as a way of transferring knowledge, it was perceived to be of less value compared to coaching and mentorship programs:

*“We used to do supportive supervision based on a checklist, but now we have changed that to coaching. Coaching is onsite based on their area of interest”* ***Round2-Facility-Ethiopia***

Some facilities shared their narratives about the approaches they used to decrease the ANC 1-4 dropout rate. Although this sharing occurred across facilities, some respondents felt that it was not easy to adapt the strategies to fit their context due to differences in geographical status and population. As such, although the national-level strategic direction could be unified, approaches to quality-of-care improvements differed from facility to facility. Respondents indicated that adoption of QoC strategies across facilities should be data-driven, however, there has not been any evidence to support the feasibility of any intervention or approach before adaption:

*“...You need to collect data that incorporates process. It should be evidence based. Being evidence based, the data that we have got on learning can fill and can correct gaps. So, the network has improved the process of quality improvement. It enhanced commitment; brought learning; mobilized resources. So, I think these are the benefits of networking”* ***Round2-National-Implementation Partner-Ethiopia***

## Discussion

The emergence of the QCN contributed to sharing and learning across member countries and their corresponding QCN facilities. Promotion of key knowledge sharing and learning drivers, development, and strengthening of mechanisms that facilitate knowledge sharing, as well as those related to data system improvement, were evident at the global, national, and facility levels.

The secretariat for the network and stakeholders at the national level led efforts to create and improve knowledge sharing platforms between countries. A number of conferences and webinars were hosted, providing access to materials on strategies and approaches within the network. Strengthening of learning collaborative session meetings, coaching, and mentorship were also evident at the national and local level. This served as evidence of the progress made by the QCN to achieve the learning strategic objective outcome of setting up mechanisms to facilitate learning and to share knowledge through a learning network.

While the network has created platforms and contributed to the promotion of learning, innovation, and sharing for quality improvement, supplementary improvements and investments in individual and organizational capacities are required to effect greater changes [15,20].

### Implementation challenges across all countries

Although most of the national-level actors, relative to facility-level actors, had actively been involved in several global-level initiatives, their attendance hardly contributed to transforming the day-to-day operations of the QCN in facilities [15]. Resources and approaches learned from other countries have not substantially descended to facility-level actors. Furthermore, there have not been a substantial number of innovations reported across the network countries; most of the learning and sharing initiatives and platforms were from broader maternal and child health initiatives, not necessarily specific to the QCN. This also made it challenging to distinguish QCN activities from routine quality improvement initiatives.

Further, implementation of quality of care network initiatives had been donor-driven [15,16]. Most of the donors providing funding in-country supported quality improvements aligned with their strategic plans and not necessarily those guided by the QCN secretariat or ministry of health. For instance, national-level respondents indicated that it was not easy to test the ideas learned because the implementation partners responsible for channeling resources had other priority areas. This is also posed as a challenge to the sustainability of network activities [21].

Mentorship, coaching, and collaborative learning sessions have been key for individual learning facilities to interface with each other and the national level to share projects and progress. However, no real adoption of these approaches has been achieved. The lack of a coherent evidence generation system to qualify the intervention process contributed to a lack of evidence to distinguish what qualifies as a working intervention, and why and how such an intervention works. Monitoring data for quality improvement was generally found to be a work in progress, with many gaps in availability and accuracy, as corroborated by our related study on the effectiveness of QCN [15].

### Limitations

Our selected study countries and respondents were purposively selected and therefore not representative of the whole population of potential respondents, this also affected results generalizability beyond the selected study countries. It was beyond the scope of this study to quantify learning, sharing, or innovation in the countries and how these affected quality of care processes and outcomes, either overall or over time. This is potentially an area for further research. It is also possible that social desirability bias [33,34] may have affected some responses; however, the anonymous nature of our study, our use of iterative inquiry over several rounds, and triangulation of data across several data sources (interviews, documents, and observations) are likely to have limited this.

It is also unclear from this evaluation how psychological empowerment and the immediate determinants of knowledge sharing intention and knowledge sharing behaviour according to Wu SY et al 2014 theory of learning and sharing are clearly linked in a multilevel health network and whether these linkages are significant. Future research could focus on this to advance understanding of how it influences sharing, learning and innovation in a health-related network.

Despite some study limitations, this evaluation of the Quality-of-Care Network (QCN), contributes knowledge to the field and has several strengths. First, the research methods demonstrate an insightful and contextually rich approach, utilizing KIIs and document reviews to capture the multilevel and multidimensional aspects of the network. The use of qualitative methods aligns well with the exploratory nature of the research topic, allowing for a deep exploratory understanding of learning and sharing mechanisms in the QCN. Further, our KII guides were not rigid across rounds of data collection; we reviewed and revised the topic guides before each round to make sure that pertinent issues from the previous round were explored during the follow-up round. Additionally, the sample selection was purposive, encompassing a diverse range of network actors from various levels of the network.

## Conclusion

Health networks are important for fostering learning, sharing, and innovation. The network promoted coordination among stakeholders, and several sharing platforms were established and meetings conducted to equip countries with implementation updates and facility quality of care approaches. Similar approaches across the study countries to sharing and learning, for example, learning collaborative sessions, were evident. However, innovations were not as apparent across countries. This is likely due to health practitioners’ tendency to mostly adopt and implement pre-existing maternal health approaches from an era preceding the QCN. However, there was evidence that the introduction of the QCN improved the functionality of learning and sharing platforms across countries.

## Supporting information

S1 Text PLoS Global Health QCN Evaluation Collection 2-page summary. (DOCX)

S2 Text QCN papers common methods section. (DOCX)

S1 Checklist Inclusivity in global research questionnaire. (DOCX)

## References

[1] Unicef. Maternal and new-born health. [cited 2024]. Available from: https://www.unicef.org/health/maternal-and-newborn-health

[2] Hug L, Alexander M, You D, Alkema L. National, regional, and global levels and trends in neonatal mortality between 1990 and 2017, with scenario-based projections to 2030: a systematic analysis. Lancet Global Health. 2019;7(6):e710–20. 31097275 10.1016/S2214-109X(19)30163-9 PMC6527519

[3] Unicef. A neglected tragedy: the global burden of stillbirths 2020; 2020 [cited 2024]. Available from: https://thedocs.worldbank.org/en/doc/845141602114822604-0090022020/original/AneglectedtragedystillbirthsIGMEreportEnglish2020.pdf

[4] UNICEF, UNFPA, WHO, World Bank. Trends in maternal mortality 2000 to 2017: estimates by WHO, UNICEF, UNFPA, World Bank Group and the United Nations Population Division: executive summary: World Health Organization; 2019 [cited 2024]. Available from: https://www.unfpa.org/featured-publication/trends-maternal-mortality-2000-2017

[5] World Health Organization. Delivering quality health services: a global imperative for universal health coverage; 2019 [cited 2023]. Available from: https://www.who.int/publications-detail-redirect/9789241513906

[6] Abraham JM, Melendez-Torres GJ. A realist review of interventions targeting maternal health in low- and middle-income countries. Womens Health (Lond). 2023;19:17455057231205687. Epub 2023/10/30 doi: 10.1177/17455057231205687 ; PMCID: PMC10617292 37899651 PMC10617292

[7] National Statistics Office. Malawi Demographic and Health Survey (MDHS) 2010. Available from: https://dhsprogram.com/pubs/pdf/fr247/fr247.pdf.

[8] Kamanga A, Ngosa L, Aladesanmi O, Zulu M, McCarthy E, Choba K, et al. Reducing maternal and neonatal mortality through integrated and sustainability-focused programming in Zambia. PLOS Glob Public Health. 2022;2(12):e0001162. Epub 2023/03/25. doi: 10.1371/journal.pgph.0001162 ; PMCID: PMC10021549 36962888 PMC10021549

[9] Jaeger FN, Bechir M, Harouna M, Moto DD, Utzinger J. Challenges and opportunities for healthcare workers in a rural district of Chad. BMC Health Serv Res. 2018;18(1):1–11. doi: 10.1186/s12913-017-2799-6 29310644 PMC5759836

[10] World Health Organization. The Network for Improving Quality of Care for Maternal, Newborn and Child Health (Quality of Care Network) [cited 2023 09]. Available from: https://www.who.int/groups/Quality-of-care-network.

[11] Larson E, Vail D, Mbaruku GM, Mbatia R, Kruk ME. Beyond utilization: measuring effective coverage of obstetric care along the quality cascade. Int J Qual Health Care. 2017;29(1):104–10. Epub 2016/12/07. doi: 10.1093/intqhc/mzw141 ; PMCID: PMC5890864 27920246 PMC5890864

[12] World Health Organisation. Monitoring framework (working document). Quality, equity, dignity: a network for improving quality of care for maternal, newborn and child health. Geneva: WHO, 2019. [cited 2024 Dec 5]. Available from: https://qualityofcarenetwork.org/knowledge-library/monitoring-framework-quality-equity-dignity-who-network-improving-quality-care.

[13] Quality of Care Network. “Network for Improving Quality of Care for Maternal, Newborn and Child Health” retrieved 20/09/2023 [cited 2024]. Available from: https://ewene.org/accelerate-maternal-and-newborn-survival/quality-of-care-network/.

[14] Lawn JE, Bhutta ZA, Ezeaka C, Saugstad O. Ending preventable neonatal deaths: multicountry evidence to inform accelerated progress to the sustainable development goal by 2030. Neonatology. 2023;120(4):491–9. Epub 2023/05/26. doi: 10.1159/000530496 ; PMCID: PMC10614465 37231868 PMC10614465

[15] Djellouli N, Shawar YR, Mwaba K, Akter K, Seruwagi G, Tufa AA, et al. Effectiveness of a multi-country implementation-focused network on quality of care: delivery of interventions and processes for improved maternal, newborn and child health outcomes. PLOS Glob Public Health. 2024;4(3):e0001751. Epub 2024/03/04. doi: 10.1371/journal.pgph.0001751 ; PMCID: PMC10911613 38437217 PMC10911613

[16] Akter K, Shawar YR, Tesfa A, Howell CD, Seruwagi G, Kyamulabi A, et al. Influences on policy-formulation, decision-making, organisation and management for maternal, newborn and child health in Bangladesh, Ethiopia, Malawi and Uganda: the roles and legitimacy of a multi-country network. PLOS Glob Public Health. 2023;3(11):e0001742. Epub 2023/11/21. doi: 10.1371/journal.pgph.0001742 ; PMCID: PMC10662733 37988328 PMC10662733

[17] Shawar YR, Djellouli N, Akter K, Payne W, Kinney M, Mwaba K, et al. Factors shaping network emergence: a cross-country comparison of quality of care networks in Bangladesh, Ethiopia, Malawi, and Uganda. PLOS Glob Public Health. 2024;4(7):e0001839. Epub 2024/07/23. doi: 10.1371/journal.pgph.0001839 ; PMCID: PMC11265678 39042649 PMC11265678

[18] Mukinda FK, Djellouli N, Akter K, Sarker M, Tufa AA, Mwandira K, et al. Individual interactions in a multi-country implementation-focused quality of care network for maternal, newborn and child health: a social network analysis. PLOS Glob Public Health. 2023;3(9):e0001769. Epub 2023/09/21. doi: 10.1371/journal.pgph.0001769 ; PMCID: PMC10513266 37733733 PMC10513266

[19] Dube A, Mwandira K, Akter K, Khatun F, Lemma S, Seruwagi G, et al. Evaluating theory of change to improve the functioning of the network for improving quality of care for maternal, newborn and child health. PLOS Glob Public Health. 2024;4(8):e0003532. Epub 2024/08/01. doi: 10.1371/journal.pgph.0003532 39088520 PMC11293647

[20] Tesfa A, Nakidde C, Akter K, Khatun F, Mwandira K, Lemma S, et al. Individual, organizational and system circumstances, and the functioning of a multi-country implementation-focused network for maternal, newborn and child health: Bangladesh, Ethiopia, Malawi, and Uganda. PLOS Glob Public Health. 2023;3(7):e0002115. Epub 2023/07/10. doi: 10.1371/journal.pgph.0002115 ; PMCID: PMC10332621 37428713 PMC10332621

[21] Lemma S, Daniels-Howell C, Tufa AA, Sarker M, Akter K, Nakidde C, et al. Opportunities to sustain a multi-country quality of care network: lessons on the actions of four countries Bangladesh, Ethiopia, Malawi, and Uganda. PLOS Glob Public Health. 2023;3(9):e0001672. Epub 2023/09/12. doi: 10.1371/journal.pgph.0001672 ; PMCID: PMC10497147 37698985 PMC10497147

[22] Quality of Care Network. Learn from other countries [2024]. Available from: https://www.qualityofcarenetwork.org/country-data.

[23] World Health Organization. Every newborn: an action plan to end preventable deaths 2014. Available from: https://iris.who.int/bitstream/handle/10665/127938/9789241507448_eng.pdf?sequence=1.

[24] Wu S-Y, Wang W-T, Hsiao M-H. Knowledge sharing among healthcare practitioners: identifying the psychological and motivational facilitating factors. Front Psychol. 2021;12:736277. doi: 10.3389/fpsyg.2021.736277 34970184 PMC8712574

[25] Kalaris K, English M, Wong G. Developing an understanding of networks with a focus on LMIC health systems: how and why clinical and programmatic networks form and function to be able to change practices: A realist review. SSM Health Syst. 2023;1:100001. Epub 2023/12/25. doi: 10.1016/j.ssmhs.2023.100001 ; PMCID: PMC10740353 38144421 PMC10740353

[26] Quality of Care Network. Videos from the network for improving quality of care for maternal, newborn and child health (quality of care network) [cited 2023 09].[cited 2024 Dec 5] Available from: https://www.youtube.com/@QualityofCareNetwork/videos (now part of https://www.youtube.com/@EWENEglobal.

[27] Health Newborn Network. Safe delivery app [cited 2024]. Available from: https://www.healthynewbornnetwork.org/resource/safe-delivery-app/.

[28] Quality of Care Network. “Digital tools to improve quality of care: Lessons from using the Safe Delivery App in Ethiopia” retrieved 20/09/2023 available from: https://www.youtube.com/watch?v=xeTwbFUQaSk

[29] Thomsen CF, Barrie AMF, Boas IM, Lund S, Sørensen BL, Oljira FG, et al. Health workers’ experiences with the Safe Delivery App in West Wollega Zone, Ethiopia: a qualitative study. Reprod Health. 2019;16(1):50. Epub 2019/05/11. doi: 10.1186/s12978-019-0725-6 31072399 PMC6506934

[30] Singh Sodha T, Grønbæk A, Bhandari A, Mary B, Sudke A, Smith LT. mHealth learning tool for skilled birth attendants: scaling the Safe Delivery App in India. BMJ Open Qual. 2022;11(Suppl 1):e001928. doi: 10.1136/bmjoq-2022-001928 ; PMCID: PMC6506934 35977730 PMC9389095

[31] Malawi Ministry of Health and Population, Maternal New-born Child and Adolescent Health Quality of Care Roadmap. 2017.

[32] Malawi Ministry of Health and Population, The Malawi MNH Standards. 2019.

[33] Bispo Júnior JP. Social desirability bias in qualitative health research. Rev Saude Publica. 2022;56:101. Epub 2022/12/15. doi: 10.11606/s1518-8787.2022056004164 ; PMCID: PMC9749714 36515303 PMC9749714

[34] Grimm P. Social desirability bias. Wiley international encyclopedia of marketing. 2010.

